# Association between pelvic organ prolapse and stress urinary incontinence with collagen

**DOI:** 10.3892/etm.2014.1563

**Published:** 2014-02-20

**Authors:** LIYING HAN, LING WANG, QIANG WANG, HELIAN LI, HU ZANG

**Affiliations:** 1 Department of Obstetrics and Gynecology, Second Hospital of Jilin University, Changchun, Jilin 130041, P.R. China; 2 Department of Orthopedics, The China-Japan Union Hospital of Jilin University, Changchun, Jilin 130033, P.R. China

**Keywords:** pelvic organ prolapse, stress urinary incontinence, collagen, ultrastructure

## Abstract

The aim of the present study was to investigate the ultrastructure and content of collagen in uterosacral ligaments and paraurethral tissues in patients with pelvic organ prolapse (POP) and stress urinary incontinence (SUI), analyzing the association between POP and collagen dysfunction. The study comprised three groups: Control, POP and POP with SUI (n=30 per group). Histological characteristics of collagen fiber were observed and the diameters were measured using light and electron microscopy to determine the Type I and Type III collagen content of the main ligament in the urethral specimens. In the POP and POP with SUI groups, observations included diffuse atrophy of smooth muscles, active fibroblast metabolism, swollen mitochondria and visible Golgi apparatus. The collagen fibril diameters in the cardinal ligaments, uterosacral ligaments and paraurethral tissues were significantly greater in the POP and POP with SUI groups compared with those in the control group (P<0.01). In addition, the expression levels of Type I and Type III collagen were significantly lower in the POP and POP with SUI groups when compared with the control group (P<0.01). In the POP with SUI group, pelvic tissues were frail, resulting in smooth muscle bundles comminuting and arranging in a disorganized pattern. Fibroblast and myoblast metabolisms were active and new microvascular cells were weak. However, the collagen fibril diameter increased. Thus, collagen and ultrastructural changes in the pelvic floor may be associated with the development of POP and SUI.

## Introduction

Pelvic floor dysfunction includes pelvic organ prolapse (POP) and stress urinary incontinence (SUI). POP is a common condition among adult vaginally parous females of all ages, with a prevalence of up to 37% (1,2). Investigations in China found that the morbidity of POP among females aged >60 years was 25% (3). Dysfunctions in extracellular matrix (ECM) content form the molecular and biochemical basis for POP. However, the exact underlying molecular and cellular mechanisms remain poorly understood. Previous studies have shown that POP and other collagen-associated disorders, including varicose veins and joint hypermobility, may have a common etiology and originate at the molecular level of collagen (4–6).

Collagen is a ubiquitous biomaterial and the main component of connective tissues that provide robustness and resilience, supporting the stability and plasticity of the vagina. Type I collagen has a thick diameter and is associated with hardness, whereas Type III collagen has a fine diameter and is associated with organizational flexibility (7).

The aim of the present study was to analyze the association between POP and collagen disorders. We hypothesized that changes in the ultrastructure and content of collagen are associated with the angiogenesis of POP. Collagen content, fiber diameter and collagen ultrastructure were investigated in the cardinal ligament, uterosacral ligament and paraurethral tissues of patients with POP. The results were compared with those of the control group in order to identify the changes in collagen metabolism in POP and to determine the association between POP and collagen changes.

## Materials and methods

### Subjects and grouping

A total of 90 patients who underwent abdominal or vaginal hysterectomy between March 2009 and March 2011 at the Department of Gynecology, Second Hospital of Jilin University (Changchun, China) were selected for the study. The patients were divided into three groups. The control group (n=30) had a median age of 60.52±6.58 years, body mass index (BMI) of 24.82±4.32 kg/m^2^, gravidity of 3.14±0.78 and parity of 1.83±0.92. The POP group (n=30) had a median age of 63.24±4.84 years, BMI of 25.66±3.56 kg/m^2^, gravidity of 5.21±0.67 and parity of 3.75±0.68. Finally, the POP with SUI group (n=30) had a median age of 63.24±4.84 years, BMI of 25.66±3.56 kg/m^2^, gravidity of 5.21±0.67 and parity of 3.75±0.68. The groups exhibited no statistically significant differences in BMI and age (P>0.05). The participants had not received hormone drugs for almost 3 months and postoperative pathology results confirmed no ectopic endometrium, uterine fibroids and ovarian function of estrogen-associated diseases, including cancer. Candidates with dysfunctional uterine bleeding, uterine myoma or endometrial premalignant lesions comprised the control group. Patients who passed the diagnostic criteria for POP and POP with SUI, according to their medical history, examination results, pressure tests and urodynamic inspection, comprised the POP and POP with SUI groups. The study was conducted with approval from the Ethics Committee of Jilin University (Changchun, China) and informed written consent was provided by all participants.

### Sample

Samples of ~500 mg cardinal ligaments, 500 mg uterosacral ligaments and 500 mg paraurethral tissues were collected from the patients who underwent an abdominal or vaginal hysterectomy. Specimen preparation for election microscopy included fixation of tissue in 2.5% glutaraldehyde in 0.1 M sodium cacodylate buffer at 4°C.

### Electron microscopy

Tissues were fixed in 2.5% glutaraldehyde and 0.1 M sodium cacodylate, and dehydrated in a propylene oxide/epoxy resin mixture (50:50). Under a JEM-1010 transmission electron microscope (JEOL, Co., Ltd., Tokyo, Japan) with a magnification of ×10,000–15,000, the morphologies and structures of the collagen fibers were observed and the diameters were measured. The samples were meshed into three groups and further divided into three sections. Ten collagen fibers from each section were observed under the microscope. The cross-section perpendicular to the long axis of collagen fibers was selected and the mean values were calculated.

### Light microscopy

Specimens for light microscopy were fixed with 10% neutral formalin and then embedded in paraffin. Micron sections were mounted on glass slides and allowed to dry at 37°C for 12 h then specimens were prepared for hematoxylin and eosin (H&E) (Solarbio Bioscience & Technology Co., Ltd., Shanghai, China) and Masson’s trichrome staining using Trichrome Stain (Masson) kit (Sigma-Aldrich Co,. LLC, St Louis, MO, USA).

### Immunohistochemistry

Antibodies against Type I and Type III collagen, Strept Avidin Biotin Complex kits and SP-9,000 kits were purchased from Boster Biological Technology, Ltd. (Wuhan, China). Results were analyzed by randomly selecting five fields of vision (magnification, ×400) for each specimen. Five images of each vision were recorded.

### Statistical analysis

Mean values were compared with the Student’s t-test or χ^2^ analysis using SSPS software, version 14.0 (SPSS, Inc., Chicago, IL, USA). Two-tailed P-values of <0.05 were considered to indicate a statistically significant difference.

## Results

### Light microscope observations

**Figure 1:**
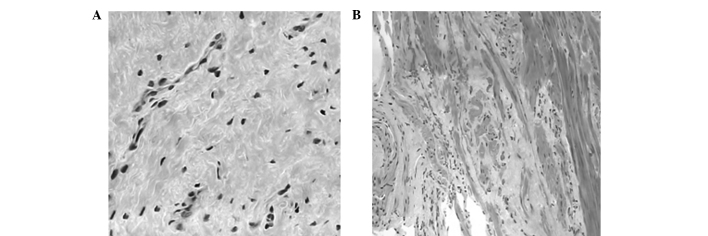
Light microscope observations of collagen and muscle fiber in the (A) POP and POP with SUI and (B) control groups (magnification, ×200). POP, pelvic organ prolapse; SUI, stress urinary incontinence.

In the POP and POP with SUI groups, Masson’s trichrome and H&E staining results showed diffuse atrophy of smooth muscles and a partial fracture. Other observations included hyaline or mucoid degeneration in sections of muscle tissues, parts of focal fibrosis distributed as an island, disorder of collagen fibers arranged in bundles, partial dissolution, swelling of microvascular endothelial cells, infiltration of peripheral inflammatory cells and irregular arrangement (Fig. 1A). In the control group, smooth muscle fibers showed a gross structure, collagen fibers were arranged in tiny bundles and microvascular cells showed normal morphology (Fig. 1B).

### Electron microscope observations

**Figure 2:**
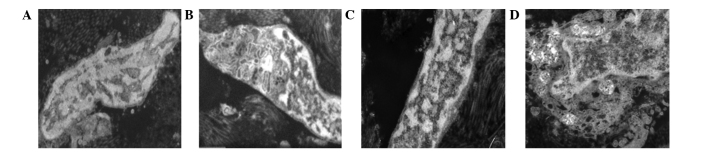
Electron microscope observations of mitochondria in the (A) POP and POP with SUI and (B) control groups. (C) Fibroblast in the POP and POP with SUI groups and (D) muscle fibroblast in POP and POP with SUI group (bar, 5 nm). POP, pelvic organ prolapse; SUI, stress urinary incontinence.

In the POP and POP with SUI groups, fibroblast metabolism was active and muscle fibroblasts were visible. Cell vacuoles in the mitochondria were swollen, the crest had decreased or disappeared and part of the mitochondrial myelin-like structure had dissolved (Fig. 2A). In addition, fibroblasts showed normal morphology, cell membrane integrity was smooth, organelles were plentiful, mitochondria were clearly visible and the ridge and microvascular endothelial cells exhibited normal morphology (Fig. 2B). The nuclear size of the fibroblast cells was relatively large and the rough endoplasmic reticulum in the cytoplasm increased. The number of mitochondria also increased and the Golgi apparatus were visible (Fig. 2C). Muscle fiber cells were saw-tooth-like, branched and ridged or had a burr-like formation. Nuclear membrane invagination, cell nuclear pyknosis and heterochromatin were increased. The diameter of collagen fibrils around the cells differed and larger gaps were visible. Immature microvascular endothelial cell aggregation was discontinuous. Cells were swollen and the cytoplasm was increased (Fig. 2D).

### Collagen fibril diameter

In the POP and POP with SUI groups, the collagen fibril diameters in the cardinal ligaments, uterosacral ligaments and paraurethral tissues were significantly greater compared with those in the control group (P<0.01; Table I).

### Expression of Type I collagen protein

**Figure 3:**
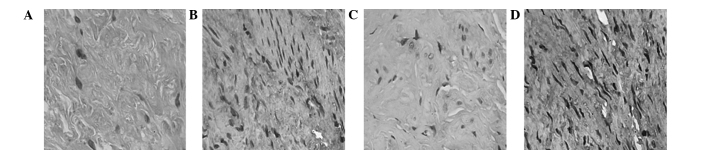
Expression of Type I collagen in the (A) POP and POP with SUI and (B) control groups. Expression of Type III collagen in the (C) POP and POP with SUI and (D) control groups (magnification, ×200). POP, pelvic organ prolapse; SUI, stress urinary incontinence.

In the control group, Type I collagen proteins in the cardiac ligaments, sacral ligaments and paraurethral tissues were asymmetrically stained. The proteins were arranged as ribbons with diffuse distribution or focal adhesion (Fig. 3A). In the POP or POP with SUI groups, Type I collagen proteins in the cardiac ligaments, sacral ligaments and paraurethral tissues were stained and distributed in unusual fractions (Fig. 3B).

The measurements from the image analysis system Image-Pro Plus 6.0 (IPP 6.0)revealed that the expression levels of Type I collagen in the cardiac ligaments of the POP group were 26.47% lower compared with the control group. However, in the POP with SUI group, the expression levels were 36.24% lower than the level in the control group (P<0.01). The expression levels of Type I collagen in the sacral ligaments of the POP group were 29.03% lower than the expression levels in the control group, but in the POP with SUI group, the expression levels were 41.89% lower compared with the control group (P<0.01). When compared with the control group, the expression levels of Type I collagen in the paraurethral tissues of the POP group were 24.43% lower, while in the POP with SUI group, the levels were 31.14% lower (P<0.01; Table II).

### Expression of Type III collagen protein

In the control group, Type III collagen proteins in the cardiac ligaments, sacral ligaments and paraurethral tissues were stained with diffuse distribution or focal adhesion (Fig. 3C). In the POP and POP with SUI groups, Type III collagen proteins in the cardiac ligaments, sacral ligaments and paraurethral tissues were marginally stained (Fig. 3D).

Measurements from the image analysis system IPP 6.0 revealed that when compared with the control group, the expression levels of Type III collagen in the cardiac ligaments of the POP group were 21.49% lower, while in the POP with SUI group the levels were 47.73% lower (P<0.01). The expression levels of Type III collagen in the sacral ligaments of the POP group were 10.84% lower compared with the control group. However, in the POP with SUI group, the expression levels were 39.35% lower compared with the control group (P<0.01). In addition, the expression levels of Type III collagen in the paraurethral tissues of the POP group were 15.74% lower than the levels in the control group, while in the POP with SUI group, the levels were 35.00% lower compared with the control group (P<0.01; Table III).

## Discussion

We hypothesized that collagen status has an important function in POP angiogenesis. The concentration of Type I and III collagen was found to significantly decrease in POP patients and active metabolism in the ultrastructure of collagen resulted in a prolapse of the pelvic floor. In the POP group, electron microscopy revealed that a number of mitochondria in fibroblasts swelled to an empty bubble, whereas others transformed into myelin-like structures. Smooth muscle bundles showed diffuse atrophy and hyaline or mucinous degeneration. In addition, collagen fibers exhibited a disorderly arrangement in partial solution.

Mitochondria are one of the most sensitive organelles to various injuries. The formation of myelin-like structures is a common sign of mitochondrial membrane damage. In addition, extreme swelling and transformation to small bubble-like structures are signs of cell necrosis. Thus, ultrastructural changes in smooth muscle cells indicate a decrease or loss in cell function, which is consistent with the results of a previous study (8). Tseng *et al* compared microarray gene expression profiles and demonstrated that an increase in the expression of mitochondrial and ribosomal protein-coding genes also increased apoptosis-associated genes in patients with POP (9). Ferrari *et al* investigated 233 females and analyzed polymorphisms at the Type I collagen Sp1 site and functional polymorphisms in the promoters of matrix metalloproteinase (MMP)-1, −3 and −9. The results demonstrated that the MMP polymorphisms possibly contribute in mediating susceptibility to POP (10), indicating that irreversible cell damage of connective tissues and smooth muscles may be the result of MMP function in patients with POP.

Electron microscopy also revealed discontinuous gathering and swelling of immature microvascular endothelial cells and increased cytoplasm in patients with POP, which were not observed in the control group. However, novel microvascular functions are unable to compensate for vascular lesions caused by inadequate blood flow and hypoxia, resulting in increased smooth muscle injury. Goepel *et al* analyzed the connective tissues in the uterine artery wall of postmenopausal females with and without POP and hypothesized that the ECM may change in response to mechanical stretching (11).

In the present study, collagen fibrils surrounding the cells were found to have varied sizes with large gaps between each fibril. In the POP group, the collagen fibril diameter was significantly greater compared with the control group, which was consistent with the results of Abramowitch *et al* (12). The pelvic floor, organized by resynthesis and degradation of elastic fibers, undergoes significant changes (13). If the pelvic floor is not remodeled, the elastic fiber network structure becomes damaged, resulting in structural and functional defects of the reproductive tract. Transforming growth factor-β pathways have been shown to be involved in ECM degradation, which is modulated by reproductive hormones and selective estrogen receptor modulators (14). Decreased HOXA11 gene expression is reportedly associated with decreased collagen and increased MMP-2 expression levels in the uterosacral ligaments of females with POP (15). In murine vaginal stromal cells, fibulin-5 inhibits the β1 integrin-dependent, fibronectin-mediated upregulation of MMP-9. Treatment of mice with β-aminopropionitrile, an inhibitor of matrix cross-linking enzymes, induces subclinical POP (16).

In the present study, the POP and POP with SUI groups exhibited significantly lower expression levels of Type I and III collagen when compared with the control group. The reduction in collagen content in the connective tissues is attributed to massive atrophy, degeneration, necrosis and fibrosis, which may also be the possible causes of POP and SUI. Zhou *et al* evaluated the elastogenic (a measure of stiffness) protein expression of Type I and Type III collagen in the vagina and demonstrated that vaginal wall tissues were stiffer in females with POP (17). Iwahashi and Muragaki found that decreased expression levels of Type III collagen have an important function in determining the physiology and structure of the uterine cervix tissues in POP (18). In connective tissues, the elastic fiber content decreases, fibers are distributed fragmentally and elastic fiber maturity manifests as declined desmosine levels and reduced expression levels of Fibulin-5 and LOX (19–21). Smooth muscle degeneration, ECM metabolism and apoptosis are inhibited in patients with POP (22). This inhibition may be caused by genetic factors that are associated with the original collagen metabolism, cell cycle or apoptosis, which result in POP. Furthermore, Pal *et al* concluded that moderate to severe POP is an independent predictor of incident spine [hazard ratio (HR), 2.61; 95% confidence interval (CI), 1.04–6.56; P=0.042] and lower arm fractures (HR, 1.87; 95% CI, 1.06–3.29; P=0.030) (23). Feola *et al* demonstrated that worsening pelvic supportive ability was negatively correlated with decreased collagen alignment (r^2^=−0.66) and mechanical properties (r^2^=−0.67) (24). Moreover, Connell *et al* reported that Type III collagen expression was directly associated with the presence of prolapse rather than age or menopausal status, and is suppressed with the use of hormone replacement therapy. If these mechanisms are elucidated, supplementary therapeutic agents with estrogens may assist in rebuilding these ligaments (25).

In conclusion, changes in collagen fibers and microvascular cells result in cell apoptosis and necrosis, causing abnormal ECM metabolism. POP is an acquired disorder of the ECM and therapies targeting matrix proteases may be efficient for preventing or ameliorating POP (7).

## Figures and Tables

**Table I:** Collagen fibril diameter of groups (nm).

Parameter	Cardinal ligament	Uterosacral ligament	Paraurethral tissues
Control group	57.72±2.61^a^	55.95±3.61^b^	43.73±2.51^c^
POP group	78.83±8.28a′^d^	77.72±7.32b′^d^	60.72±5.29c′^d^
POP with SUI group	82.23±8.49a″^d^	85.28±7.31b″^d^	63.39±5.34c″^d^
t-value	a:a′ 9.333	b:b′ 10.575	c:c′ 11.424
	a:a″ 10.837	b:b″ 14.247	c:c″ 13.219
P-value	a:a′ <0.01	b:b′ <0.01	c:c′ <0.01
	a:a″ <0.01	b:b″ <0.01	c:c″ <0.01

a Collagen fibril diameter of cardinal ligament in control group.

a′ Collagen fibril diameter of cardinal ligament in POP group.

a″ Collagen fibril diameter of cardinal ligament in POP with SUI group.

b Collagen fibril diameter of uterosacral ligament in control group.

b′ Collagen fibril diameter of uterosacral ligament in POP group.

b″ Collagen fibril diameter of uterosacral ligament in POP with SUI group.

c Collagen fibril diameter of paraurethral ligament in control group.

c′ Collagen fibril diameter of paraurethral ligament in POP group.

c″ Collagen fibril diameter of paraurethral ligament in POP with SUI group.

d P<0.01, vs. control group.

In the POP and POP with SUI groups, the collagen fibril diameter of the cardinal ligament, uterosacral ligament and paraurethral tissues was significantly greater compared with the control group (P<0.01). POP, pelvic organ prolapse; SUI, stress urinary intolerance.

**Table II:** Expression of Type I collagen in all groups (OD).

Groups	Cardiac ligament	Sacral ligament	Paraurethral tissue
Control (n=30)	124.56±5.39	123.74±5.65	116.57±5.22
POP (n=30)	91.59±3.28^a^	87.83±3.87^a^	88.10±3.90^a^
POP with SUI (n=30)	79.42±6.12^a^^b^	71.91±4.86^a^^b^	80.28±4.76^a^^b^
F-value	346.27	423.71	255.63

a P<0.01, vs. control group;

b P<0.01, vs. POP group.

POP, pelvic organ prolapse; SUI, stress urinary incontinence.

**Table III:** Expression of Type III collagen in all groups (OD).

Groups	Cardiac ligament	Sacral ligament	Paraurethral tissue
Control (n=30)	126.06±4.83	115.31±5.53	122.44±4.17
POP (n=30)	98.97±6.66^a^	102.84±5.17^a^	103.17±4.80^a^
POP with SUI (n=30)	65.90±4.11^a^^b^	69.94±4.22^a^^b^	79.59±3.83^a^^b^
F-value	355.55	218.04	287.62

a P<0.01, vs. control group;

b P<0.01, vs. POP group.

POP, pelvic organ prolapse; SUI, stress urinary incontinence.

## References

[1] Swift S, Woodman P, O’Boyle A (2005). Pelvic Organ Support Study (POSST): the distribution, clinical definition, and epidemiologic condition of pelvic organ support defects. Am J Obstet Gynecol.

[2] Kerkhof MH, Hendriks L, Brölmann HA (2009). Changes in connective tissue in patients with pelvic organ prolapse - a review of the current literature. Int Urogynecol J Pelvic Floor Dysfunct.

[3] Zhou Y, Ling O, Bo L (2013). Expression and significance of lysyl oxidase-like 1 and fibulin-5 in the cardinal ligament tissue of patients with pelvic floor dysfunction. J Biomed Res.

[4] Lammers K, Lince SL, Spath MA (2012). Pelvic organ prolapse and collagen-associated disorders. Int Urogynecol J.

[5] Yucel N, Usta A, Guzin K (2013). Immunohistochemical analysis of connective tissue in patients with pelvic organ prolapse. J Mol Histol.

[6] Cho HJ, Jung HJ, Kim SK, Choi JR, Cho NH, Bai SW (2009). Polymorphism of a COLIA1 gene Sp1 binding site in Korean women with pelvic organ prolapse. Yonsei Med J.

[7] Budatha M, Roshanravan S, Zheng Q (2011). Extracellular matrix proteases contribute to progression of pelvic organ prolapse in mice and humans. J Clin Invest.

[8] Boreham MK, Wai CY, Miller RT, Schaffer JI, Word RA (2002). Morphometric analysis of smooth muscle in the anterior vaginal wall of women with pelvic organ prolapsed. Am J Obstet Gynecol.

[9] Tseng LH, Chen I, Lin YH, Chen MY, Lo TS, Lee CL (2010). Genome-based expression profiles study for the pathogenesis of pelvic organ prolapse: an array of 33 genes model. Int Urogynecol J.

[10] Ferrari MM, Rossi G, Biondi ML, Viganò P, Dell’utri C, Meschia M (2012). Type I collagen and matrix metalloproteinase 1, 3 and 9 gene polymorphisms in the predisposition to pelvic organ prolapse. Arch Gynecol Obstet.

[11] Goepel C, Johanna Kantelhardt E, Karbe I, Stoerer S, Dittmer J (2011). Changes of glycoprotein and collagen immunolocalization in the uterine artery wall of postmenopausal women with and without pelvic organ prolapse. Acta Histochem.

[12] Abramowitch SD, Feola A, Jallah Z, Moalli PA (2009). Tissue mechanics, animal models, and pelvic organ prolapse: a review. Eur J Obstet Gynecol Reprod Biol.

[13] Edwall L, Carlström K, Fianu Jonasson A (2008). Markers of collagen synthesis and degradation in urogenital tissue and serum from women with and without uterovaginal prolapse. Mol Hum Reprod.

[14] Petros PE (2012). Re: Alterations in connective tissue metabolism in stress incontinence and prolapse: B. Chen and J. Yeh J Urol 2011; 186: 1768–1772. J Urol.

[15] Ma Y, Guess M, Datar A (2012). Knockdown of Hoxa11 in vivo in the uterosacral ligament and uterus of mice tesults in altered collagen and matrix metalloproteinase activity. Biol Reprod.

[16] Ewies AA, Al-Azzawi F, Thompson J (2003). Changes in extracellular matrix proteins in the cardinal ligaments of post-menopausal women with or without prolapse: a computerized immunohistomorphometric analysis. Hum Reprod.

[17] Zhou L, Lee JH, Wen Y (2012). Biomechanical properties and associated collagen composition in vaginal tissue of women with pelvic organ prolapse. J Urol.

[18] Iwahashi M, Muragaki Y (2011). Decreased type III collagen expression in human uterine cervix of prolapse uteri. Exp Ther Med.

[19] Klutke J, Ji Q, Campeau J (2008). Decreased endopelvic fascia elastin content in uterine prolapse. Acta Obstet Gynecol Scand.

[20] Söderberg MW, Byström B, Kalamajski S, Malmström A, Ekman-Ordeberg G (2009). Gene expressions of small leucine-rich repeat proteoglycans and fibulin-5 are decreased in pelvic organ prolapse. Mol Hum Reprod.

[21] Jung HJ, Jeon MJ, Yim GW, Kim SK, Choi JR, Bai SW (2009). Changes in expression of fibulin-5 and lysyl oxidase-like 1 associated with pelvic organ prolapse. Eur J Obstet Gynecol Reprod Biol.

[22] Takacs P, Nassiri M, Gualtieri M, Candiotti K, Medina CA (2009). Uterosacral ligament smooth muscle cell apoptosis is increased in women with uterine prolapse. Reprod Sci.

[23] Pal L, Hailpern SM, Santoro NF (2011). Increased incident hip fractures in postmenopausal women with moderate to severe pelvic organ prolapse. Menopause.

[24] Feola A, Abramowitch S, Jones K, Stein S, Moalli P (2010). Parity negatively impacts vaginal mechanical properties and collagen structure in rhesus macaques. Am J Obstet Gynecol.

[25] Connell KA, Guess MK, Chen H, Andikyan V, Bercik R, Taylor HS (2008). HOXA11 is critical for development and maintenance of uterosacral ligaments and deficient in pelvic prolapse. J Clin Invest.

